# The Asylums of Italy, Germany, and France

**Published:** 1857-10-01

**Authors:** John T. Arlidge

**Affiliations:** Member of the Royal College of Physicians of London; formerly Resident Medical Officer of St. Luke's Hospital, London


### Art. IX.—THE ASYLUMS OF ITALY, GERMANY, AND FRANCE.

### NOTES OF A VISIT MADE IN THE YEAR 1855.

BY JOHN T. ARLIDGE, M.B., A.B. (LOND.),

Member of the Royal College of Physicians of London; formerly Resident Medical Officer of St. Luke's Hospital, London.

(*Continued from page 547.*)

*Asylum of Florence.*—The Royal Asylum or hospital for the insane of Florence, Leghorn, and other departments of Tuscany, is situated within the walls of Florence, near the Porta St. Gallo, and is a dependency of the great general hospital, the "Arcispedale di Santa Maria Nuova." It accommodates both sexes, and its inmates are divided into two classes of pensioners, or paying patients, and one of paupers.

It is under the superintendence of two physicians, one of whom, Dr. Bini, is the director, and the other, Dr. Cardini, the "adjoint." They are assisted in their duties by an apothecary and by several "internes." The building, originally designed for a monastery, is ill-adapted for an asylum in its internal structural arrangements, and still more so in its want of any sufficient airing-courts, exercise-ground, and gardens. It was converted to its present use about seventy years since; its defects are recognised by its medical officers, and a building in lieu of it has been proposed in the country, but many years will probably elapse before this desirable and much needed removal is carried out. Indeed, the political position of the Government of Tuscany and the want of means are in themselves formidable obstacles to the prosecution of any large plans for the amelioration of the condition of the insane. All sorts of difficulties encompass even the minor attempts at improvement and reform in the existing asylum; some originating from its dependence both in its administration and funds upon the general hospital; others from the non-medical authorities who specially govern it, for the signatures of some four or five persons are needed to guarantee the smallest expenditure for any improvement or alteration, and therefore, as may well be supposed, it is a sufficiently arduous task to persuade so many independent authorities of the purpose and utility of any plan proposed. The existing structure is rightly fitted to accommodate not more than 350 patients, but the increasing demand for admission for several years has caused it to be crowded with above 400, and at times with more than 500. Some three months before my visit, in June, 1855, this crowding had reached a maximum, when the cholera broke out among the inmates, and carried off 106, of whom two-thirds were females.

And it is worthy of remark, that this devastation was on the most crowded and the worst built side. No new case had occurred for three weeks; the disease was chiefly confined to this hospital, and did not spread among the inhabitants of the city.

The building is tolerably regular in construction, consisting of two divisions, with a chapel between them. Each section forms a hollow square of about 100 feet, around a court or exercising ground. The general elevation is of two stories; the walls of stone, and well constructed. On one side the building and the chapel is the public street; behind there is a garden, occupying above an acre, cultivated as a kitchen and pleasure garden by the patients; and on the outer side of one division is another plot of ground, partly paved, but generally laid down in grass, and planted with an avenue of acacias. The enclosed square courts are paved, and have neither shelter nor seats for the patients.

On the ground floor the rooms open on one side a wide corridor; but on the floor above, the corridor is central, with rooms on each side. The dining and day-rooms for each division occupy the ground floor, together with the bath-room, the kitchen, the visiting, or general amusement-room, and some dormitories and cells for epileptic, refractory, and dirty patients. The floors of the corridors and bath-rooms, and those on the ground floor generally, are of stone; in the rooms above, of tiles.

Most of the windows look into the central courts; they are all barred with vertical iron bars. The single rooms have each a window on one side of the door, of about 18 inches square, and 4½ feet from the ground, filled with crossed iron bars. This serves as an aperture for inspection. Beneath it is a recess, closed by a door opened from the corridor, intended for a cupboard to keep the patient's clothes in when taken off at night.

The rooms constructed for a single inmate are spacious, and afford accommodation for two-thirds of the patients; but it is much to be deplored that, owing to the crowded state of the hospital, they are for the most part, except indeed in the wards for the refractory, occupied by two patients. The evils and dangers of this proceeding do not seem to have struck the medical directors of the Italian asylums, since, as we have already noted, the same plan is pursued at Genoa. The prisonlike character, too decidedly conveyed by the narrow limits of the asylum, by its bare walls and barred windows, is much aggravated by the open iron doors of many of the rooms and of the corridors, and by similar doors, not much improved in appearance by the substitution of wooden for iron bars, on the staircase landings, and at some other parts.

The dormitories are few in number, but contain a considerable number of beds. On the female side there are three, holding each 20 patients. In one of these the windows are placed between 9 and 10 feet above the floor, and it consequently has a heavy, gloomy aspect, more suited to a prison than to an asylum. After the outbreak of the cholera among the females, one dormitory and some small chambers, where the disease particularly prevailed, were given up, and in lieu of them, a large ward was taken into use, capable of accommodating 50 inmates.

The bedsteads in general use are of iron, and of the common form. Those occupied by epileptics are furnished with sides, to prevent their falling out during a paroxysm. No injury from these sides has been witnessed.

Except for dirty and destructive patients, the bedding consists of a paillasse, a flock-bed, sheets, a woollen quilt or blanket, with a cotton coverlet. For those of dirty and destructive habits, similar beds were in use, but instead of a flock-bed and paillasse, the latter only was afforded them. No special beds were provided for paralytics; indeed, the adoption of water and of spring-beds may be said to be confined to British asylums. Certainly the number of paralytics in the Italian asylums I visited was small, and the peculiarities and requirements of their condition less understood and appreciated than in this country. Bed-sores were looked upon as unavoidable, attention to changing the position being the chief measure relied on for their prevention and relief.

The sleeping-rooms were clean, in good order, and generally free from smell; their walls coloured, mostly white-washed. The dormitories and corridors are imperfectly lighted by small lamps during the night, and an attendant keeps watch. No padded-room existed, but seclusion in a darkened chamber for a brief period was resorted to as a means to calm and repress patients under maniacal excitement.

A common dining-room and a common sitting or day-room belonged to each division. The latter was partially separated into three apartments—for the convalescent clean and orderly, for the refractory, and for the demented. The dining-room was a long room, bare and uncomfortable in appearance, as likewise was the sitting-room. No attempt to cover and vary the bare whitewashed wall with pictures or otherwise was made; no provision of settees or comfortable chairs for the weak, no books or amusements were to be seen, to relieve the monotonous, unoccupied, and dreary life of the unfortunate inmates—that is, those of the indigent class, whose accommodation has hitherto been particularly spoken of.

The dining-tables had a cleanly look, their top being formed of a variegated marble slab, defended by a wooden frame against fracture or other damage. The meals are eaten with forks and spoons, knives not being allowed, chiefly from being unnecessary, since the food is broken up by stewing, or by its preparation in the shape of soup. Were it not so, knives would be little patronized, the custom of the poor being to employ their fingers and the direct action of their jaws to effect the severance of any victuals calling for division before deglutition.

On the female side there is a needle-room, in which a considerable number of the women are daily employed; but for the men no occupation, except, indeed, for a few in the small garden, is provided. The advantages of workshops are thoroughly recognised by the physicians, but their efforts to make the ruling powers think with them have hitherto been all in vain.

There is a small common or visiting-room, in which patients receive visits from their friends, and where occasional dances are held, for a small and select number, in which the two sexes join.

The pensioners enjoy certain advantages both with respect to diet and accommodation; when quiet, the windows of their bed-rooms are furnished with curtains, they have a better made bed-stead, superior bedding, a table, and generally drawers, with a chair or two, necessary minor articles of furniture, and a few ornaments. They dine together in a common room; their table is covered with a cloth, and each has his napkin for use. Their sitting-room is also common, and is provided with a piano, a small collection of books, and a few articles for amusement. It has, however, the fault of being too small.

The pensioners are, moreover, dressed variously, according to the means and wishes of their friends, whilst the indigent, on the other hand, wear generally a uniform dress provided by the asylum.

The bath rooms are good; there is one on each side of the building. The baths themselves are capacious—7 feet by 3 feet, and $2\frac{1}{2}$ feet in depth, and made of marble. Water, both hot and cold, enters by an opening at the bottom, which also serves for the discharge. The water-cocks of the bath supply-pipes are out of the reach of the patients, and require a key to turn them. The boiler-room is on the female side, adjoining the bath-room, and affords a constant supply of hot water. The douche is sometimes employed as a means of repression, but more frequently a small stream of water is let trickle upon the head when the patient is placed in a warm bath. Shower-baths, of the English fashion, are here, as elsewhere on the Continent, not in vogue; a sort of substitute is sometimes resorted to by screwing a "rose" upon the end of a douche-pipe.

No system of bathing, as a means of cleanliness, is pursued, and lavatories with soap and towels are among the things unthought of.

The ventilation of the building is left to the natural currents of air by the doors, windows and corridors; and, excepting a stove in the large dormitory used as an infirmary, no means of warming are in existence. Certainly, the climate of this part of Italy renders any general plan of heating a public institution unnecessary, yet I am persuaded that, for a short time in winter the presence of a stove in the eating and sitting rooms would be both agreeable and advantageous to the inmates. For it will be readily granted that the sufferers from mental disease, by reason of their usually feeble and ofttimes sluggish circulation, require the cherishing influence of warmth even more than the sane.

The kitchen is of good size and well managed. It has one of those excellent Italian cooking stoves, built up in its centre, with hot-plates and small boilers on all sides, rendering the processes of cooking simple and easily performed. A scullery adjoins the kitchen. The provision is apportioned by the cook and assistants, and then distributed in the dining-rooms to the patients.

The chapel is conveniently placed between the two sections of the building. It is divided into two portions, one for each sex, chiefly by the high-altar, and a screen extended from it on each side, so that those seated before and behind it cannot see each other. Each sex enters into its allotted half by a door opening immediately into the respective corridor. The chapel is well-kept and ornamented in the manner usual to Roman-Catholic places of worship. Altogether it must afford to the unfortunate lunatics an agreeable and useful change from the dull wards which they generally occupy.

The "moral treatment" can necessarily be but very indifferently carried out in an asylum so destitute of the requisite means; where an excessive number is crowded within its walls; where the opportunities for employment and amusement are so few and incomplete, and where the number of attendants is so small that thirty patients or upwards are allotted to the charge of each. (The whole number of attendants, I was told, did not exceed fourteen.) It will, therefore, surprise no one to learn that mechanical restraint, of much severity, is often resorted to, and held to be a necessary adjunct to the treatment of the insane. It is employed for the furious to restrain them from harming others, for the suicidal to preserve them from self-injury, for the restless at night to confine them in their beds, and for the destructive to impede their propensities. Having these various objects in view, sufficient occasions are found by over-tasked, ill-educated, and ill-paid attendants for its use. Yet, notwithstanding the aid of all this coercion, an English superintendent would be ill enough pleased to have an equally noisy population, untidy and ragged in dress, and prone to destruction and mischief.

In fact, not a few were ragged and disorderly in dress, and some bare-footed. But further, the apparent liberty to relieve the bladder and to spit at pleasure about the corridors, was another practice very revolting to our advanced ideas of asylum propriety and discipline.

Although compelled to notice these defects in the Florence asylum, it is, at the same time, due to the able and well-intentioned medical staff to state that they cannot be held entirely accountable for this state of things. For, as we set out by observing, they are not free and independent agents in conducting the asylum, but the servants of an impracticable non-medical board; and further, they are under the influence of the prevalent doctrine of their country of the necessity of restraint, and have had no means of learning those multitudinous expedients for lessening and abolishing it, which are so familiar that they seem to come almost intuitively to the mind of an English asylum superintendent. For example, the use of strong dresses for tearers, or of peculiarly fastened boots for those who will keep none on, has never suggested itself. Moreover, the position of the asylum within the walls of a large city, its circumscribed area, the absence of means for employment and amusement, and the consequent entailment of an indolent, monotonous life on the inmates—rather calculated to foster the delusions of many of them than to eradicate them, are clearly adducible as circumstances extenuating the blame which would otherwise attach to the medical officers on account of the unsatisfactory condition of the institution. To these impediments to the existence of a well-conducted asylum should be added another, which must exercise a potent effect, and probably is at the root of all the rest, viz., the deficiency of funds, or more correctly, perhaps, the niggardly manner in which they are doled out for the purposes of the institution, especially when any alteration or improvement is projected.

Still we should regret to see money expended on the present building, since no expenditure could ever render it a fitting habitation for the care and treatment of the insane. On the contrary, what is needed, is an entirely new building, erected in the open country, replete with every necessary internal arrangement, and surrounded with ample fields and gardens. And no difficulty could be encountered in finding a suitable site for such a new institution in this delightful Florentine territory, where nature lavishes every charm of scenery and vegetation to solace and cheer the broken spirit and to calm the troubled breast.

To revert, however, to details of moral management as they exist. Mechanical coercion, as already intimated, is largely resorted to; for, although limited as far as thought practicable and advantageous, twenty-five patients were, at the time of my visit, placed under it. This was in the day-time, for at night the number is very much increased, restraint being resorted to for those thought suicidal or dangerous to others, as well as for the restless and destructive.

Restraint is accomplished by chairs, on the model of those for which St. Luke's Hospital once claimed the merit of invention, but slightly improved upon by the Florentine mechanics. According to precedent, they were made of stout wood, had the usual hole in the seat, to add to their other useful purposes that of a water-closet; on this the patient was fixed by sundry belts, —varying in number and strength according to the supposed exigencies of the case, by the closed foot-board in front, and by the strong wooden flap falling down over the knees, and serving at will either as the trencher on which to place the victuals, or to repose the elbows when the sufficient amount of liberty was accorded to the hands. The improvement on the old model consisted in the foot-board (in front of the legs) being made oblique instead of vertical, so that a little more space was allowed to stretch out the legs. This advantage was, as most will think, unfortunately counteracted by another contrivance,— no doubt the pride of its inventor, viz., the perforation of the foot-board by holes at proper distances, so that straps might be passed through them to attach the ankles of the occupant of the chair, and in that way prevent the somewhat unpleasant drumming with the feet with which he might be disposed to entertain his neighbours. Nor was the luxury of leather padding to the chair denied to those who might not duly appreciate the *dolce far niente* of sitting on a sort of night-commode by the hour or day, and improperly abuse the chair by blows, whether of arms or head.

Lastly, care was taken by placing three or four of them in a row, that no patient should be alone in the privilege of occupying one of these chains.

Thus much has been said about the restraint-chair, since its use still lingers in several Continental asylums, and some of our readers, especially the younger portion, may never have had an opportunity of seeing that ingenious contrivance in this country.

Besides the chair, other instruments of restraint were in vogue; viz., the hand-muff, camisole, and belts. The muff in general form resembled that heretofore used in English asylums, having, however, certain peculiarities of construction. It consisted of a piece of stout leather, large enough to envelope the hands and wrists, the edges being locked together. Previously to placing this round the hands, these were fastened together by a belt, acting like a hand-cuff. At times, hand-cuffs, or manacles made of leather, were resorted to in lieu of an ordinary belt; at others, the hands were fastened to a belt worn round the waist, or enclosed in thick leathern gloves. These various contrivances, including the camisole, for exercising mechanical coercion, admitted, by their number, of several variations and combinations which it is unnecessary to specify.

The belts, muffs, and gloves were extended to night as well as day use; and, in addition, not a few cases were found which it was deemed necessary to secure in bed, by means of straps to some part of the bedstead.

This asylum of Florence afforded me an opportunity of seeing a barbarous piece of mechanism, contrived in the last generation by, we are sorry to believe, an English physician, intended both to detect feigned insanity and to cure the actual disease. This apparatus was the "whirling-chair." A small room was occupied by it and the mechanism to turn it. It may be found described and figured in one or more English books on insanity, to which we must refer those curious to learn more about it.

Latterly, I was happy to hear it had been totally disused, and trust its existence within the asylum will never suggest its re-employment. If it be worth while to preserve it as a curiosity, it would be much better to find it a place in some museum of antiquities, among the devices of the ancient Inquisition.

Dr. Cardini informed me that in some two or three instances this whirling-chair had appeared of service, but that in almost all he believed it to be mischievous.

A few more particulars will complete our account of this asylum. Soup, as elsewhere in Italy, constitutes the staple article of diet, and is taken usually at every meal, made with or without meat. Bread is also largely eaten, and is of very good quality and white. Meat, boiled usually, but sometimes roasted, enters into the dietary every day in the week, Fridays excepted. The œsophageal tube is very rarely resorted to in forced feeding.

Comparatively few cases of general paralysis or of epilepsy occur in the establishment—a fact well shown by the statistical tables which we append.

TABLE I.

*Movement of the Population of the Asylum from the* 1*st of March*, 1841, *to the* 31*st of December*, 1851, *inclusive.*

| | | Men. | Women. | Total. |
|---|---|---|---|---|
| Remaining, March 1st, 1844 | | 177 | 210 | 387 |
| Admitted | | 1215 | 1051 | 2266 |
| Discharged | cured | 523 | 404 | 927 |
| | relieved | 116 | 103 | 219 |
| | uncured | 86 | 80 | 166 |
| | found not insane | 94 | 74 | 168 |
| Dead | | 357 | 352 | 709 |
| Remaining, Dec. 31st, 1851 | | 215 | 250 | 465 |

### TABLE II.

*Movement of the Population in the Two Years* 1852 *and* 1853.

| | Men. 1852. | Men. 1853. | Women. 1852. | Women. 1853. | Total. 1852. | Total. 1853. |
|---|---|---|---|---|---|---|
| Remaining, January 1st | 215 | 226 | 250 | 247 | 465 | 473 |
| Admitted | 172 | 164 | 147 | 132 | 319 | 296 |
| Discharged: cured | 56 | 47 | 47 | 64 | 103 | 116 |
| Discharged: relieved | 16 | 14 | 23 | 11 | 39 | 25 |
| Discharged: uncured | 21 | 15 | 26 | 9 | 47 | 24 |
| Discharged: not insane | 21 | 19 | 8 | 6 | 29 | 25 |
| Dead | 47 | 54 | 46 | 36 | 93 | 90 |
| Remaining, 31st Dec. 1853 | 228 | 241 | 247 | 253 | 473 | 494 |

The three following statistical tables refer to certain questions less fully illustrated in asylum reports in general—viz., to the numbers admitted, discharged, and dead, in different months and seasons of the year :—

### TABLE III.

*Admissions between* 1844 *and* 1853, *inclusive.*

| | Men. | Women. | Total. | | Men. | Women. | Total. |
|---|---|---|---|---|---|---|---|
| In January | 101 | 94 | 195 | In July | 178 | 165 | 343 |
| February | 120 | 83 | 203 | August | 156 | 137 | 293 |
| March | 120 | 95 | 215 | September | 114 | 109 | 223 |
| April | 152 | 102 | 254 | October | 110 | 120 | 230 |
| May | 154 | 124 | 278 | November | 112 | 76 | 188 |
| June | 173 | 156 | 329 | December | 80 | 81 | 161 |

### TABLE IV.

*Discharges between* 1844 *and* 1853, *inclusive.*

| | Men. | Women. | Total. | | Men. | Women. | Total. |
|---|---|---|---|---|---|---|---|
| In January | 69 | 49 | 118 | In July | 96 | 104 | 200 |
| February | 49 | 57 | 106 | August | 83 | 108 | 191 |
| March | 73 | 52 | 125 | September | 126 | 74 | 200 |
| April | 75 | 63 | 138 | October | 113 | 82 | 195 |
| May | 87 | 63 | 150 | November | 92 | 83 | 175 |
| June | 80 | 63 | 143 | December | 94 | 60 | 154 |

### TABLE V.

*Deaths between* 1844 *and* 1853, *inclusive.*

| | Men. | Women. | Total. | | Men. | Women. | Total. |
|---|---|---|---|---|---|---|---|
| In January | 46 | 48 | 94 | In July | 33 | 30 | 63 |
| February | 24 | 35 | 59 | August | 37 | 30 | 67 |
| March | 36 | 29 | 65 | September | 32 | 36 | 78 |
| April | 26 | 19 | 45 | October | 45 | 43 | 88 |
| May | 27 | 31 | 58 | November | 60 | 44 | 104 |
| June | 34 | 40 | 74 | December | 56 | 54 | 110 |

Table III. shows a rapid rise in the number of admissions with the onset of the hot weather of summer in both sexes. The three hottest months, June, July and August, exhibit the highest, and November, December and January, the lowest number. The next Table conveys no very distinct facts; the most prominent is that the largest number was discharged in the latter summer and the autumn months, between July and November. Still the value of this fact is but small, since no information is given respecting the condition of those discharged, or the proportion of cured and uncured. Lastly, the table of deaths shows clearly the fatal effects of the cold of winter upon the insane, and affords a sufficient contradiction to the ancient tradition of their immunity from atmospheric variations, and particularly from cold. The three coldest months, November, December and January, stand first in order, and next after these, the hot summer and the variable autumnal months, when, besides mere atmospheric heat and cold, other morbid influences start into activity, especially in warmer climates such as Italy.

### TABLE VI.

*Proportion of Cures and of Deaths between* 1850 *and* 1853, *inclusive.*

| | 1850. | | 1851. | | 1852. | | 1853. | |
|---|---|---|---|---|---|---|---|---|
| | Men. | Wom. | Men. | Wom. | Men. | Wom. | Men. | Wom. |
| Remaining, January 1st, 1850 | 226 | 277 | 238 | 289 | 215 | 250 | 226 | 247 |
| | 503 | | 527 | | 465 | | 473 | |
| Admitted . . . . | 191 | 142 | 183 | 162 | 172 | 147 | 164 | 132 |
| | 333 | | 345 | | 319 | | 296 | |
| Cured . . . . . | 64 | 56 | 86 | 74 | 56 | 47 | 47 | 64 |
| | 120 | | 160 | | 103 | | 111 | |
| Uncured and discharged as not insane . . | 59 | 26 | 62 | 64 | 58 | 57 | 47 | 26 |
| | 85 | | 126 | | 115 | | 74 | |
| Died . . . . . | 56 | 48 | 58 | 63 | 47 | 46 | 54 | 36 |
| | 104 | | 121 | | 93 | | 90 | |
| Remaining, December 31st, 1853 . . . . | 238 | 289 | 215 | 250 | 226 | 247 | 241 | 253 |
| | 527 | | 465 | | 473 | | 494 | |
| Ratio of Cures per cent. to Admissions . . . . | 33·5 | 39·4 | 46·9 | 45·7 | 32·5 | 31·9 | 22·5 | 48·4 |
| | 36·3 | | 46·3 | | 32·2 | | 37·5 | |
| Ratio of Cures to entire Population . . | 15·3 | 13·3 | 20·4 | 16·1 | 14·4 | 11·8 | 12·5 | 16·8 |
| | 14·3 | | 18·3 | | 13·1 | | 14·4 | |
| Ratio of Deaths to Admissions . . . | 29·3 | 33·8 | 31·6 | 38·8 | 27·3 | 31·9 | 32·9 | 27·2 |
| | 31·2 | | 35·7 | | 29·1 | | 30·4 | |
| Ratio of Deaths to entire Population . . | 13·4 | 11·4 | 13·7 | 13·9 | 12·1 | 11·5 | 13·9 | 9·4 |
| | 12·4 | | 13·8 | | 11·8 | | 11·7 | |

TABLE VII.

*Form of Mental Disorder of those admitted between* 1850 *and* 1853, *inclusive.*

| | Males. | Females. | Total. |
|---|---|---|---|
| Idiocy . . . | 31 | ... 26 | ... 57 |
| Dementia . . . | 134 | ... 132 | ... 266 |
| Stupidity . . . | 33 | ... 22 | ... 55 |
| Monomania . . | 45 | ... 38 | ... 83 |
| Melancholia . . | 123 | ... 113 | ... 236 |
| Mania . . . | 141 | ... 139 | ... 280 |
| Acute delirium . . | 8 | ... 5 | ... 13 |
| Moral insanity . . | 18 | ... 25 | ... 43 |
| Paralysis of insane . | 53 | ... 7 | ... 60 |
| Epilepsy . . . | 46 | ... 33 | ... 79 |
| Simulated insanity . | 20 | ... 1 | ... 21 |
| Delirium tremens . | 4 | ... 0 | ... 4 |
| Febrile delirium with miliaria | 3 | ... 3 | ... 6 |
| Insanity not proved to exist | 61 | ... 39 | ... 100 |

Under the head of Monomania, M. Bini has referred all cases of partial insanity (delirium), not associated with timidity, sadness, or despair,—the characters of melancholia.

TABLE VIII.

*Form of Mental Disease of those remaining in the Asylum, December* 31, 1853.

| | Men. | Women. | Total. |
|---|---|---|---|
| Idiocy . . . . | 18 | ... 25 | ... 43 |
| Dementia . . . . | 115 | ... 123 | ... 238 |
| Stupidity . . . . | 8 | ... 9 | ... 17 |
| Monomania . . . . | 14 | ... 13 | ... 27 |
| Melancholia . . | 16 | ... 8 | ... 24 |
| Mania . . . . | 29 | ... 46 | ... 75 |
| Moral insanity . . | 0 | ... 2 | ... 2 |
| Paralytics . . . | 6 | ... 3 | ... 9 |
| Epileptics . . . | 32 | ... 16 | ... 48 |
| Convalescent . . | 3 | ... 7 | ... 10 |
| Found not insane . | 0 | ... 1 | ... 1 |

Of these, 335 are reckoned as quiet patients, and 159 as excited or refractory; 169 are stated to be dirty in habits, and 280 capable of work. The proportion of quiet and refractory cases in the two sexes is about equal, but that of females of dirty habits slightly exceeds that of males. The number of pensioners of the first class is only 18, and of the second class 24; the bulk of the asylum population being made up of the pauper or indigent class, 494 in number.

### TABLE IX.

*Of the Social Condition of the Patients, December,* 1853.

| | Men. | Women. | Total. |
|---|---|---|---|
| Single | 188 | 145 | 333 |
| Married | 44 | 83 | 127 |
| Widowed | 9 | 25 | 34 |
| Total | 241 | 253 | 494 |

### TABLE X.

*Ages of those admitted between* 1850 *and* 1853, *inclusive.*

| | Males. | Females. | Total. |
|---|---|---|---|
| Under 10 years | 7 | 4 | 11 |
| From 10—20 | 61 | 47 | 108 |
| 20—30 | 191 | 156 | 347 |
| 30—40 | 160 | 111 | 271 |
| 40—50 | 143 | 118 | 261 |
| 50—60 | 65 | 80 | 145 |
| 60—70 | 51 | 49 | 100 |
| 70—80 | 28 | 14 | 42 |
| 80—90 | 4 | 4 | 8 |
| Total | 710 | 583 | 1293 |

### TABLE XI.

*Physical Causes of the Mental Disorder in Cases admitted between* 1850 *and* 1853, *inclusive.*

| | Males. | Females. | Total. |
|---|---|---|---|
| Defect of cerebral development | 36 | 21 | 57 |
| Traumatic injuries of skull | 28 | 5 | 33 |
| Sanguineous congestion and apoplexy | 10 | 9 | 19 |
| Epilepsy | 58 | 49 | 107 |
| Exposure to sun | 21 | 3 | 24 |
| Abuse of wine and spirits | 112 | 22 | 134 |
| Abuse of tobacco | 28 | — | 28 |
| Venereal excess | 19 | 1 | 20 |
| Prolonged use of mercury | 17 | 3 | 20 |
| Prolonged use of quinine | 2 | 1 | 3 |
| Masturbation | 30 | 8 | 38 |
| Pregnancy | — | 5 | 5 |
| Childbirth | — | 15 | 15 |
| Dysmenorrhœa and amenorrhœa | — | 22 | 22 |
| Change of life | — | 2 | 2 |
| Lactation | — | 12 | 12 |
| Typhoid fever | 4 | 5 | 9 |
| Intermittent fever | 3 | 2 | 5 |
| Miliaria | 3 | 9 | 12 |
| Suppression of habitual discharges | 8 | 3 | 11 |
| Decrepitude | 8 | 11 | 19 |
| Pellagra | 20 | 16 | 36 |
| Hereditary predisposition | 172 | 159 | 331 |

### TABLE XII.

*Moral Causes of the Mental Disorder in Cases admitted between* 1850 *and* 1853, *inclusive.*

| | Males. | Females. | Total. |
|---|---|---|---|
| Want | 51 | 29 | 80 |
| Mental distress, not well defined | 66 | 66 | 132 |
| Political changes | 23 | 3 | 26 |
| Fear | 30 | 35 | 65 |
| Love, disappointed | 26 | 47 | 73 |
| Jealousy | 6 | 15 | 21 |
| Offended self-love | 12 | 1 | 13 |
| Disappointment | 7 | 1 | 8 |
| Outrage on modesty | — | 3 | 3 |
| Domestic indifference—ennui | 30 | 44 | 74 |
| Domestic troubles | 13 | 23 | 36 |
| Scruples of conscience | 29 | 37 | 66 |
| Failure in business—reverses of fortune | 41 | 13 | 54 |
| Gaming | 5 | — | 5 |
| Imprisonment | 12 | — | 12 |
| Excessive study and novel reading | 4 | 1 | 5 |
| Excessive joy | — | 1 | 1 |
| Unascertained | 78 | 98 | 176 |

### TABLE XIII.

*Occupations of Patients admitted between* 1850 *and* 1853, *inclusive.*

| Men. | | Women. | |
|---|---|---|---|
| Peasants and labourers | 247 | Peasants and labourers | 177 |
| Independent persons | 43 | Occupied in domestic labour | 119 |
| Shopkeepers | 34 | Cooks, tailors, shoebinders | 60 |
| Shoemakers | 15 | Servants | 49 |
| Soldiers | 15 | Independent | 26 |
| Scriveners, or lawyers | 21 | Weavers and needlewomen | 25 |
| Hatters | 12 | Straw-hatmakers | 23 |
| Agents—(*facchini*) | 13 | Shopkeepers | 6 |
| Sawyers | 12 | Nuns | 4 |
| Bricklayers | 12 | Of other occupations 2, 3, or 4 of each | 16 |
| Musicians | 10 | Without occupation | 78 |
| Seamen | 8 | | |
| Barbers | 7 | | |
| Coachmen | 6 | | |
| Engravers | 8 | | |
| Priests | 10 | | |
| Monks | 7 | | |
| Cooks | 10 | | |
| Advocates | 5 | | |
| Of all other trades, some 3, 4, or 5 of each | 136 | | |
| Employment not known | 35 | | |

### TABLE XIV.

*Causes of Death from* 1850 *to* 1853, *inclusive.*

| | Males. | Females. | Total. |
|---|---|---|---|
| Epileptic fits | 6 | 5 | 11 |
| Acute delirium | 6 | 1 | 7 |
| Sanguineous congestion of head | 11 | 17 | 28 |
| Encephalitis | 6 | 2 | 8 |
| Cerebral hæmorrhage | 11 | 5 | 16 |
| Serous effusion in head | 43 | 32 | 75 |
| Pneumonia | 11 | 14 | 25 |
| Pulmonary gangrene | 6 | 3 | 9 |
| Phthisis | 16 | 15 | 31 |
| Cardiac disease | 4 | 8 | 12 |
| Pleuritic effusion | 6 | 3 | 9 |
| Hydropericardium | 0 | 2 | 2 |
| Asphyxia | 1 | 1 | 2 |
| Diaphragmatic hernia | 1 | 0 | 1 |
| Tabes mesenterica | 7 | 2 | 9 |
| Chronic diarrhœa | 31 | 25 | 56 |
| Cirrhosis | 0 | 3 | 3 |
| Ascites | 2 | 0 | 2 |
| Softening of spleen | 0 | 1 | 1 |
| Peritonitis | 14 | 15 | 29 |
| Dysentery | 4 | 1 | 5 |
| Chronic cystitis | 1 | 0 | 1 |
| Miliaria | 1 | 3 | 4 |
| Hydræmia | 6 | 6 | 12 |
| Scurvy | 1 | 6 | 7 |
| Marasmus from fasting or anæmia | 12 | 14 | 26 |
| Typhoid fever | 5 | 5 | 10 |
| Pellagra | 2 | 2 | 4 |

### TABLE XV.

*Relapses among those admitted from* 1850 *to* 1853, *inclusive.*

| | Males. | Females. | Total. |
|---|---|---|---|
| Admitted for the first time | 516 | 421 | 937 |
| Re-admitted after relapse | 109 | 100 | 209 |
| Re-admitted after discharge as not cured | 54 | 45 | 99 |
| Re-admitted after discharged as "not found insane" | 31 | 17 | 48 |

Upon a review of these several tables, a few facts worthy of attention will strike the reader. The number of males admitted exceeds that of females, which would seem to show a greater proclivity to insanity among the former. Again, if in the relative number of the two sexes there is a similar preponderance of males over females in the population of Tuscany to that found in England, then the proclivity of the male sex appears still more pronounced. But another fact comes out which would not *a priori* be anticipated—viz., that the female population of the asylum, notwithstanding the smaller number of the sex admitted, exceeds that of the male, and augments in an increasing ratio year by year. To what is this attributable? It is to the fact that the ratio of deaths and of cures to the entire population has been smaller among the women than the men, consequently the former have accumulated in successive years above the latter in number, as chronic or incurable cases. This less curability of insane females and their less mortality are facts opposed to the generally received statements, as well as to the statistics of most asylums, and would seem to indicate the operation of some special causes at the Florence asylum.

The mortality during the first year after admission, by reason of the acute and recent nature of many of the cases, will necessarily be larger than in subsequent years. At Florence the mortality in relation to the number of admissions appears very high—viz., about thirty-one per cent., and yet, as a table supplied by M. Bini shows, it is more favourable than at several other Italian, German, and French asylums. Calculated upon the whole number of inmates, *i. e.*, those remaining at the commencement of any year added to those admitted in the course of that year, it is reduced to between twelve and thirteen per cent.

On referring to Table VII., showing the form of malady of those admitted in the course of four years, it is clearly seen how large a number of cases, hopelessly incurable or having but slight chances of cure, is received, composed of idiots, demented, epileptic, and paralytic patients—a number which must very materially interfere with the proportion of cures effected. From a comparison of the number of cured, considered according to the form of their malady, with the number of each variety existing, at the end of the year, M. Bini thus represents their curability per cent.—

| | | |
|---|---|---|
| Dementia . . . . | 7 to 8 | per cent. |
| Stupidity . . . . | 22 to 29 | ,, |
| Monomania . . . | 30 to 36 | ,, |
| Melancholia . . . | 39 to 46 | ,, |
| Mania . . . . | 34 to 42 | ,, |
| Moral insanity . . . | 65 to 68 | ,, |

The smaller ratio of cures in cases of mania than might be expected from the statistics and observations of most physicians (which show that when acute and treated early they are recoverable in a large proportion), M. Bini accounts for by the circumstance that it is the practice to retain lunatics under observation in the general hospitals for a time before sending them to the asylum, during which some few get well, whilst in most the chances of cure are lessened by the detention and absence of appropriate treatment.

The relative fatality of the several forms of mental disorder is thus expressed per cent. :—

| | |
|---|---|
| Idiocy . . . . | 9 to 12 per cent. |
| Dementia . . . . | 24 ,, |
| Stupidity . . . . | 29 ,, |
| Monomania . . . | 4 to 7 ,, |
| Melancholia . . . | 11 to 17 ,, |
| Mania . . . . | 10 to 12 ,, |
| Paralysis of insane . . | 60 to 72 ,, |
| Epilepsy, with insanity . | 20 to 25 ,, |

These calculations, however, both with respect to the curability and to the mortality of the several phases of mental alienation are merely approximative, since they require to be made not only for one or two, but for a series of years, on the plan pursued of taking the number of cases of each particular form existing at the commencement of any particular year, adding the number of cases admitted within the year, and then calculating from the number of deaths the proportion per cent. of the mortality under that form.

The prevalence of the general paralysis of the insane is greater in Tuscany than most writers on the Asylums of Italy have represented. M. Bini states it to be about eight per cent. among the men and one per cent. among the women in his establishment.

The proportion of relapses to the admissions is between fifteen and sixteen per cent., or, when compared with the cures, thirteen per cent.

From the table (X.) exhibiting the ages on admission during four years, the largest number of cases appear to occur between the twentieth and thirtieth years, *i.e.*, at a decennial period earlier in life than that shown by the statistics of France and England—viz., that between 30 and 40. However, if it be taken into consideration that the whole number of persons between 30 and 40, in any population, is smaller than that between 20 and 30, then although the recorded number of insane patients be absolutely smaller, yet, relatively to the population, the proportion is greater even in Tuscany, and still greater in this country, where of the actual number of admissions the largest belong to the later decennial epoch. Again, the high number of admissions at the more advanced period, between 40 and 50, falling little short of that seen between 30 and 40, should be remarked as indicating even a somewhat larger proclivity to mental disease.

Owing to the many difficulties of obtaining accurate histories of cases, and of eliminating apparent or assigned causes, often rather the symptoms of the malady, the statistics of the causes are always very uncertain and of comparatively small value. From those extracted from M. Bini's reports, physical causes are shown to preponderate greatly.

The tables of the occupations and conditions in life of those admitted show that by far the greatest number come from the peasant and labouring classes, a fact which might be anticipated from their large number relative to the entire population of a country. Still there are not sufficient data to show how far as a class they are prone to insanity proportionately to other classes, unless statistics were prepared to exhibit their relative number to the whole population.

On reviewing the assigned causes of death as set forth in the tables, a few only appear to preponderate at all largely over the rest in the long category. Phthisis, which figures so extensively in English reports among the causes of death in the insane, occupies quite a second-rate position in the annals of the Florentine asylum. During a period of four years, and in an average population of nearly 500, only 31 of 405 deaths from all causes are attributed to that disease. This proportion would appear to be less than that occurring among the citizens of Florence, and is accounted for by M. Bini by the circumstance that a large number of the patients in the asylum are country labourers, little predisposed to pulmonary consumption. Serous effusion on the head occupies the highest rank among the assigned causes of death; but, in our opinion, this abnormal condition is very rarely a primary cause of death, but a secondary effect of debility and exhaustion, and instead of enumerating the 75 deaths as instances of serous effusion, we feel that they should rather count as deaths by exhaustion. The 12 deaths attributed to hydræmia should likewise, in all probability, enter into the same category, the watery condition of the blood being the predisposing cause to serous effusion on the head.

Lesions of the abdominal viscera stand next among the causes of death, chronic diarrhœa having ended fatally in 56 cases. To peritonitis 29 deaths are referred in the course of the four years; however, it was very unequally prevalent in these several years. Thus no death from it occurred in 1850, whilst 15 happened in 1851, in 1852, 10, and in 1853, 4. The fatal outbreak in 1851 M. Bini attributes to the sudden variations of temperature which characterized that year, and against which no sufficient protection was offered by the clothing of the patients.

The large number of unmarried among the inmates of the Florence Asylum is very striking, being somewhat more than double the number of married and widowed together. The value of this fact is much lessened by our not knowing the statistics of the population at large in reference to their social condition. The large number (169) of patients dirty in their habits reflects much on the management of the asylum, being more than a third of the whole number of inmates. Great allowance must, however, be made for the medical officers, from the almost insuperable difficulties under which they labour from an over-crowded, ill-built, and ill-arranged asylum, without space for out-door employment or amusement, and with a most incomplete staff of attendants.

From the number, 280, considered capable of work, M. Bini presses upon the Florentine government the necessity and the advantages of providing means for their employment; and we trust his appeal will not be in vain.

We perceive that the Government has proposed to convert the Villa di Castel-Pulci, situated on a most agreeable hill, not far from Florence, to the purposes of an additional asylum to relieve the over-crowding of the present one of St. Boniface, in the city. For this intended improvement we must be thankful; but at the same time, we are bound to express our conviction that this conversion of an unsuitable building to the purposes of an asylum, will be attended with great cost, and be always encumbered with numerous defects and disadvantages, which the construction of a special building would at once obviate.

At Sienna is another Tuscan asylum, intended for the departments of Sienna and Grossetano. It is called the Hospice of St. Nicholas, and is situated within the walls of the city, close to the Porta di Roma. The building it occupies was formerly a monastery. I did not have the opportunity of visiting it, but understood from Dr. Cardini, that it was moderately well conducted, although, as a building, ill-adapted, and destitute of any ground for the exercise and amusement of the inmates.

At a short distance from Lucca is the asylum of Fregionaja, standing on the side of a pleasantly sloping hill. Since I saw it only in passing along the road, I am enabled to state nothing further respecting it, than that it is a three-storied modern building, having a centre and two wings, with a small space behind it, laid out in airing-courts, the whole walled in. The surrounding country is very beautiful, and no exception could be taken to its position, but I heard that it was in a very indifferent state.

